# Information Accumulation over Time in Monkey Inferior Temporal Cortex Neurons Explains Pattern Recognition Reaction Time under Visual Noise

**DOI:** 10.3389/fnint.2016.00043

**Published:** 2017-01-12

**Authors:** Ryosuke Kuboki, Yasuko Sugase-Miyamoto, Narihisa Matsumoto, Barry J. Richmond, Munetaka Shidara

**Affiliations:** ^1^Graduate School of Comprehensive Human Sciences, University of TsukubaTsukuba, Japan; ^2^Human Informatics Research Institute, National Institute of Advanced Industrial Science and TechnologyTsukuba, Japan; ^3^Laboratory of Neuropsychology, National Institute of Mental HealthBethesda, MD, USA; ^4^Faculty of Medicine, University of TsukubaTsukuba, Japan

**Keywords:** inferior temporal cortex, information theory, pattern recognition, visual noise, behavioral latency, rhesus monkey

## Abstract

We recognize objects even when they are partially degraded by visual noise. We studied the relation between the amount of visual noise (5, 10, 15, 20, or 25%) degrading 8 black-and-white stimuli and stimulus identification in 2 monkeys performing a sequential delayed match-to-sample task. We measured the accuracy and speed with which matching stimuli were identified. The performance decreased slightly (errors increased) as the amount of visual noise increased for both monkeys. The performance remained above 80% correct, even with 25% noise. However, the reaction times markedly increased as the noise increased, indicating that the monkeys took progressively longer to decide what the correct response would be as the amount of visual noise increased, showing that the monkeys trade time to maintain accuracy. Thus, as time unfolds the monkeys act as if they are accumulating the information and/or testing hypotheses about whether the test stimulus is likely to be a match for the sample being held in short-term memory. We recorded responses from 13 single neurons in area TE of the 2 monkeys. We found that stimulus-selective information in the neuronal responses began accumulating when the match stimulus appeared. We found that the greater the amount of noise obscuring the test stimulus, the more slowly stimulus-related information by the 13 neurons accumulated. The noise induced slowing was about the same for both behavior and information. These data are consistent with the hypothesis that area TE neuron population carries information about stimulus identity that accumulates over time in such a manner that it progressively overcomes the signal degradation imposed by adding visual noise.

## Introduction

Generally humans and other primates can identify a visual stimulus accurately from a brief glance. The physical properties that are combined to make objects distinguishable in natural scenes encompass a wide variety of physical properties: size, orientation, color, contrast, movement, and clarity. Thus, the brain integrates information rapidly from all of these physical properties (Richmond and Optican, 1987; Eskandar et al., 1992a,b; Ito et al., 1995; Logothetis and Sheinberg, 1996; Tanaka, 1996; Sugase et al., 1999; Riesenhuber and Poggio, 2002; Dicarlo et al., 2012; Hirabayashi et al., 2013; Martin and Schröder, 2013; Pagan et al., 2013). In primates including humans visual perception is tolerant to variations that degrade the image or make it more ambiguous (Bussey et al., 2003; Jones et al., 2003; Emadi and Esteky, 2013; Komura et al., 2013). Shidara and Richmond (2005) showed that as the amount of visual noise degrading a visual stimulus increases, monkeys maintain their performance in identifying the stimulus correctly by taking progressively more time to make a decision using black-and-white patterns which have been shown to elicit differential responses in inferior temporal cortex neurons (Richmond et al., 1987; Eskandar et al., 1992a,b). Here, using the same task (Shidara and Richmond, 2005), we studied what happens to stimulus selective information in area TE of inferior temporal cortex when the stimulus is degraded by random dot visual noise, e.g., the raindrops scattered on the glass window, or snow and fog.

The signals carried by neurons in area TE are of particular interest for object recognition because area TE is considered as a late stage in processing visual information used to recognize complex objects. We found that the amount of visual noise superimposed on the stimuli was directly related to the rate as which stimulus-related information accumulated in the responses of the TE neurons we recorded.

## Materials and methods

All experiments and procedures were carried out in accordance with the Guidelines for the Care and Use of Laboratory Animals as published by the National Research Council of the U.S. National Academy of Sciences and adopted by the National Institutes of Health. The experiments were approved by the Animal Care and Use Committee of the National Institute of Mental Health.

### Animal preparation and behavioral paradigm

**Figure 1:**
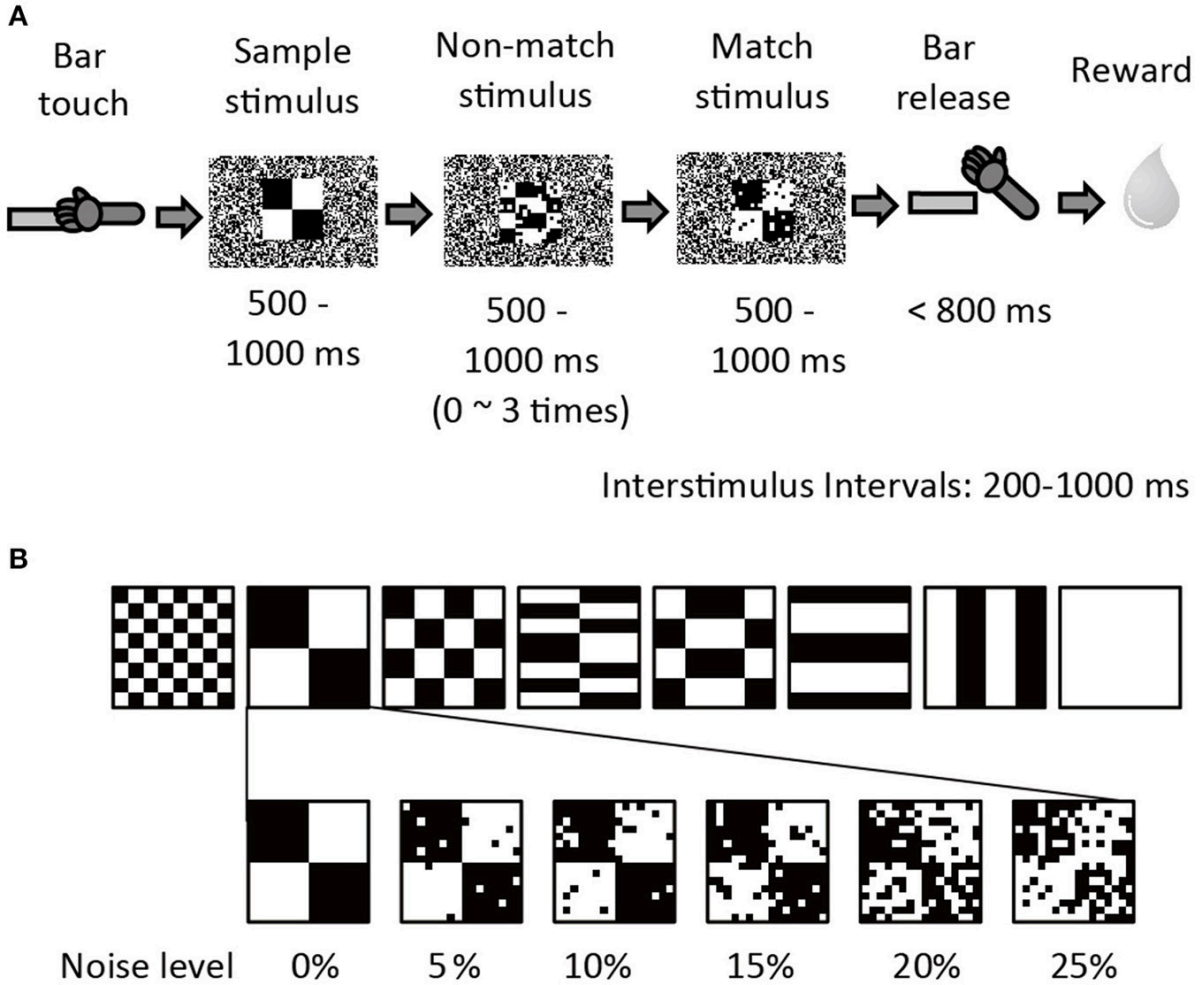
**Sequential Delayed Match to Sample (DMS) task with visual noise. (A)** The procedure of sequential DMS task. The example shows a 10% noise level. **(B)** Six noise conditions (0, 5, 10, 15, 20, and 25%) were prepared for each of eight stimulus patterns. All stimulus and noise patterns were shown in Shidara and Richmond (2005).

Two adult 5–7 kg male rhesus monkeys (*Macaca mulatta*; monkey G & S) were used to collect the behavioral and neuronal data. The experimental apparatus and behavioral paradigm have been previously described in Shidara and Richmond (2005). The monkeys were trained to perform sequential delayed match-to-sample (DMS) task (Figure 1A). When the monkey touched the bar in the chair, a small white fixation spot (0.83 × 0.83°) appeared in the center of the monitor for 500 ms. Then a series of 2–5 stimuli appeared in succession. The first stimulus was a sample, and the remaining 1–4 stimuli were test stimuli (non-match or match stimuli). To obtain a reward the monkey was required to release the bar between 150 and 800 ms when the test stimulus was a repeat of the sample, that is, the match appeared. Each stimulus was 10.0 × 10.0 degrees, and appeared at the center of the screen for a randomly chosen interval of 500–1000 ms. The interstimulus interval (ISI) was 200–1000 ms. During the interstimulus intervals, the fixation spot reappeared. The visual stimuli (same as in Shidara and Richmond, 2005) were 8 black-and-white patterns using 48 × 48 video monitor pixels. A 1-pixel black-and-white random dot background covered the whole monitor screen. To add visual noise, groups of 3 × 3-pixel dots were reversed from white to black or black to white for the non-match and match stimuli so that the number of white-black reversed pixels was 5, 10, 15, 20, or 25% (Figure 1B). The noise pattern was changed among several noise sets on an irregular rotating cycle of 1 day to a few weeks to prevent the monkeys from memorizing the noise pattern itself in the training session. The noise pattern was fixed during the recording session. The sample stimulus was always presented without noise.

After the monkeys learned to perform the task at the overall correct rate of more than 80%, a head holder and a recording cylinder were fixed to the skulls during an aseptic surgical procedures carried out under isoflurane anesthesia. The cylinder for single unit recording was attached at anterior 17 mm (monkey G) or 16 mm (monkey S). A scleral magnetic search coil was implanted under Tenon's capsule to measure eye position (Robinson, 1963; Judge et al., 1980). The fixation window was 10° or 20°. Neuronal recordings began 1 week or later after surgery.

### Neuronal recording

Single units were recorded while the monkeys performed the DMS task. A hydraulic microdrive was mounted on the recording cylinder, and tungsten microelectrodes with impedance of 1.5–1.7 MΩ (MicroProbe, Clarksburg, MD) were used through a stainless steel guide tube. Experimental control and data collection were performed by a Hewlett-Packard Vectra 486/33, using a real-time data acquisition program (Hays et al., 1982) adapted for the QNX operating system. Single units were discriminated according to spike shape and amplitude by calculating principal components using an IBM PC compatible microcomputer (Abeles and Goldstein, 1977; Gawne and Richmond, 1993). We used MR imaging to confirm that our recordings were taken from the ventral bank of inferior temporal cortex (area TE) (Saunders et al., 1990). The depths of recording sites were calculated from the lengths of the guide tubes and electrodes. The recording depths were further confirmed by calculating backwards from the depth at which the electrode struck the dura at the bottom of some penetrations.

### Data analysis

Data with fewer than 6 trials for any stimulus were discarded. A neuron was regarded as responsive if averaged spike counts between 50 and 500 ms after the onset of the sample stimulus were significantly higher than the average spike counts between 725 and 275 ms before the appearance of the fixation point (intertrial interval, ITI) for at least one of the 8 sample stimuli (*p* < 0.05, *t*-test).

For behavioral performance, error rate and reaction time averaged across all recording sessions were calculated. Error rate was defined as the ratio of the number of error trials to the number of all trials. Reaction time was defined as the time from the onset of match stimuli to bar release.

Data analysis were performed using “R” statistical computing environment (R Foundation for Statistical Computing, R Development Core Team, 2008).

### Onset latency analysis

To analyze the effect of visual noise on the neuronal responses, we measured the onset latency of neuronal responses for each noise level. First, the spike density function (SDF), which is an estimate of spike probability over time, was constructed for each individual response in each noise level by replacing each spike with a Gaussian pulse, σ = 10 − 30 ms (convolving the spike train with a Gaussian pulse). These were averaged at each millisecond (**Figure 4**). The onset latency was defined as the first time after 50 ms after the match onset when the firing rate exceeded the mean ± 2SD of the ISI response during 50 ms immediately before the match onset.

### Information theoretic analysis

Mutual information is a measure of the interdependence of two stochastic variables (Optican and Richmond, 1987). The mutual information between the neural responses and the eight stimulus patterns was calculated.

$$I\left(S;R\right)={\left(\sum_{S}P(s|r)log\left[\frac{P(s|r)}{P(s)}\right]\right)}_{r}$$

where *I(S;R)* is the mutual information between the stimulus sets, *S*, and the neural responses *R*. *S* is the average over each stimulus, and *s* is the stimulus related to neural response ***r***. *P(s|**r**)* is the probability of *s* given ***r***, i.e., the conditional probability of the stimulus being selected on the basis of the neural response ***r***. *P(s)* is the a priori probability of the stimulus, which is determined by the experimenter as the experimental settings. Obtaining an accurate estimate of the mutual information, *I(S;R)*, requires an accurate estimate of *P(s|**r**)*. We have done this using a neural network to carry out a nominal regression of the experimental condition on the neural response with care to avoid over-fitting. The analysis performed by the neural network is similar to logistic regression (Kjaer et al., 1994; Heller et al., 1995; Shidara et al., 1998; Shidara and Richmond, 2004; Inaba et al., 2013). As the response codes, spike counts from the match stimulus interval were used. Because there were almost equal numbers of trials, the maximum amount of information that could have been transmitted about the visual stimulus, that is the entropy, is log_2_8 or 3 bits. To examine the temporal change in the information accumulation, we calculated the information through an expanding time window beginning 50 ms after the stimulus onset and incremented in 8 ms steps up to 500 ms.

## Results

From 37 recorded neurons (19 neurons from monkey G, 18 neurons from monkey S), data of 13 neurons that had enough number of trials for each noise level were analyzed (7 neurons from monkey G, 6 neurons from monkey S, see Section Materials and Methods).

### Behavioral performances

**Figure 2:**
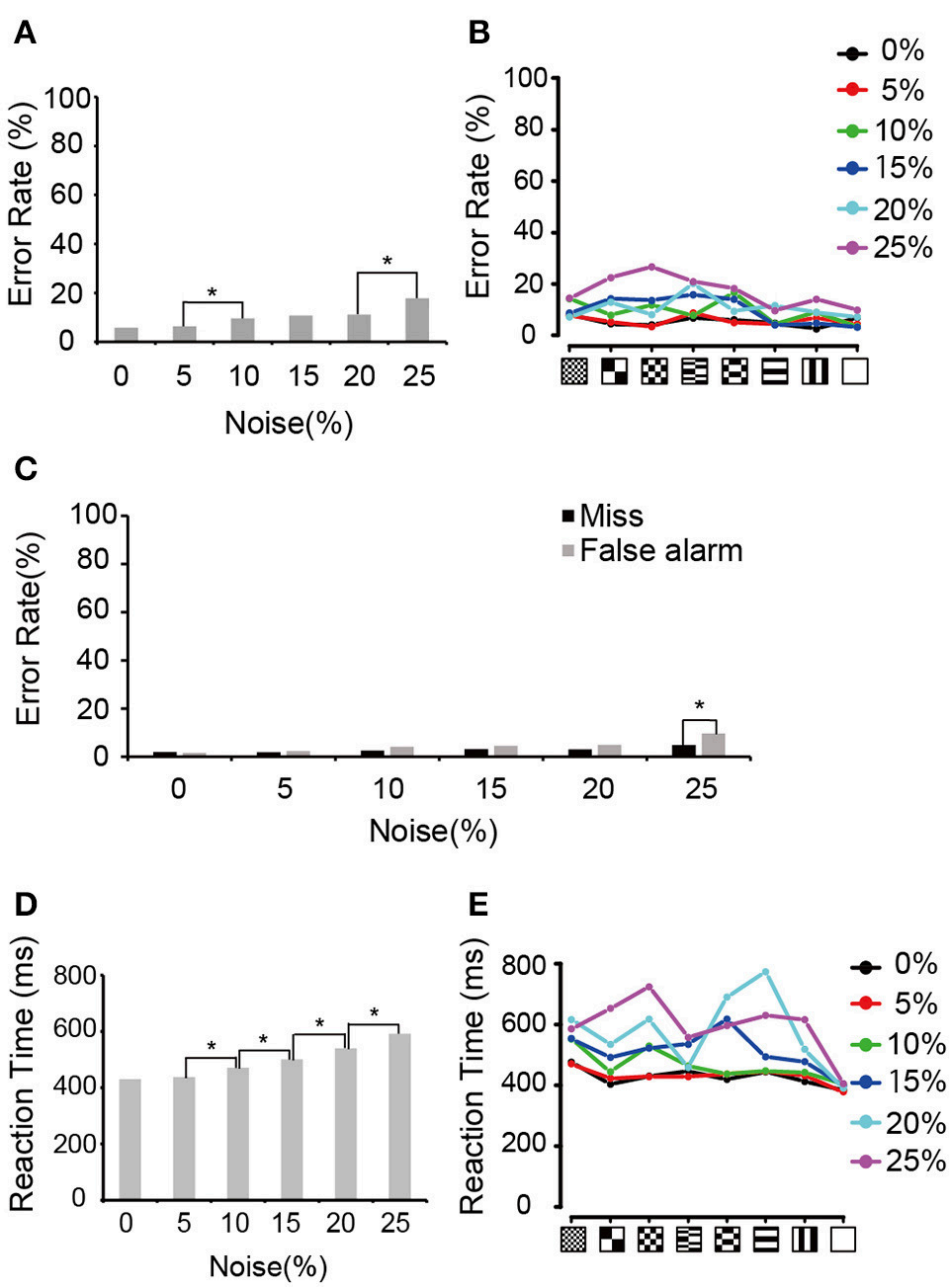
**Behavioral performances. (A)** Error rates averaged between two monkeys. Asterisks indicate the significant difference between adjacent noise levels (Chi-squared test; ^*^p < 0.05, *p*-values adjusted for multiple comparisons using the FDR procedure). **(B)** Error rates for each pattern. Black, red, green, blue, aqua blue, and purple curves indicate the error rates in 0, 5, 10, 15, 20, and 25% noise levels, respectively. **(C)** Errors divided into false alarm (bar release error in non-match presentation) and miss (not releasing the bar during match presentation). Asterisk indicates a significant difference between false alarm and miss in the same noise level (Chi-squared test; ^*^p < 0.05). **(D)** Median reaction time averaged between two monkeys. Asterisk indicates a significant difference in the reaction times between adjacent noise levels (Wilcoxon rank sum test; ^*^p < 0.05, *p*-values adjusted for multiple comparisons using the FDR procedure). **(E)** Median reaction time for each pattern. The same convention as in **(B)**.

We first analyzed the error rate and the reaction times during neuronal recording periods. The monkeys generally kept low error rates at all visual noise levels (Figure 2A), although there was significant difference between 5 and 10% noise levels and between 20 and 25% noise levels (Chi-squared test, *p* < 0.05, *p*-values adjusted for multiple comparisons using the Benjamini and Hochberg false discovery rate (FDR) procedure). A similar trend was observed in the error rates for each pattern (Figure 2B). In Figure 2C, the error rate was divided into “false alarm” (bar release error during the presentation of the non-match) and “miss” (not releasing the bar during match presentation). There were no significant differences between those errors except in 25% noise level. The median reaction time for bar release when the match stimulus appeared increased as the amount of visual noise increased except between 0 and 5% noise levels (Figure 2D, Wilcoxon rank sum test, *p* < 0.05, *p*-values adjusted for multiple comparisons using the FDR procedure). This tendency was similar to that reported previously by Shidara and Richmond (2005). Figure 2E shows the median reaction time for each pattern.

### Neuronal responses to match stimuli

**Figure 3:**
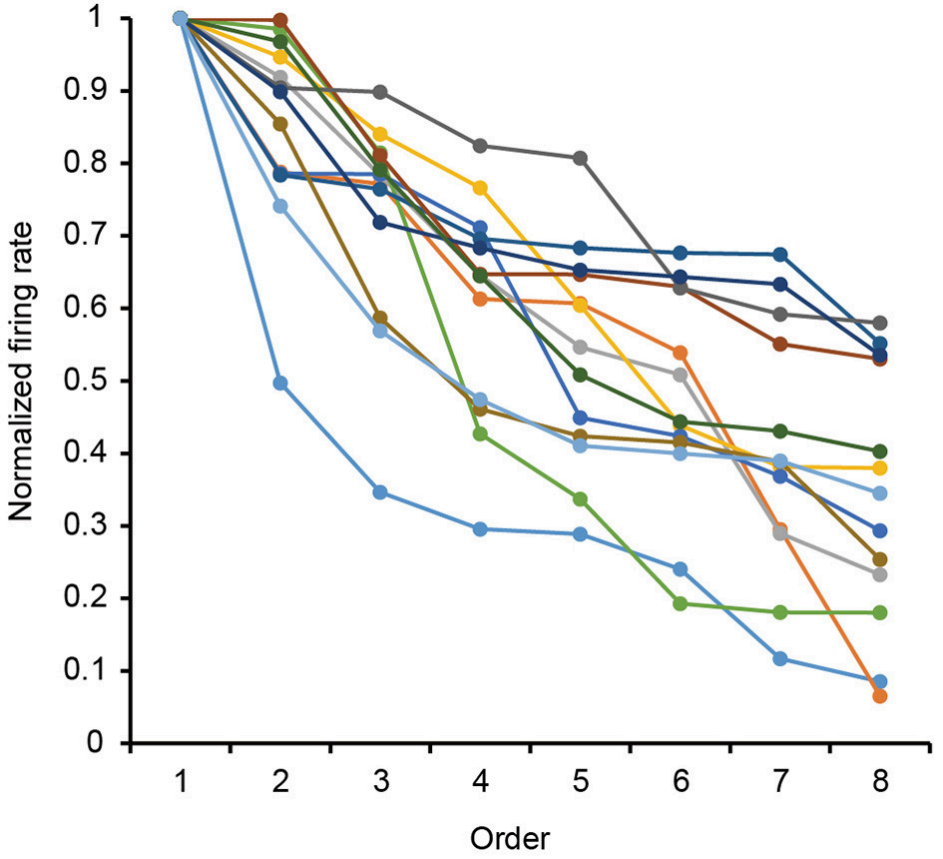
**Rank-ordered normalized response to the eight match stimuli for 13 analyzed neurons**. Firing rates for each stimulus pattern in 0% noise level were normalized by their max firing rate in 0% noise level. Each curve shows the data from each neuron.

**Figure 4:**
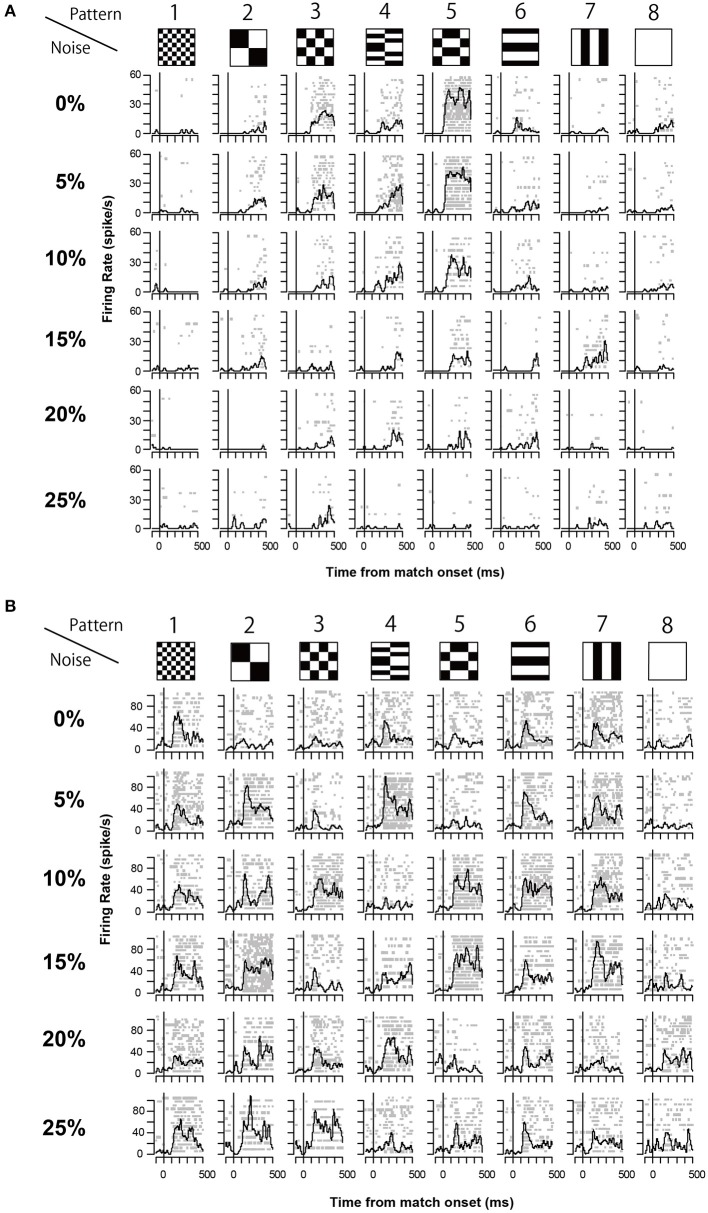
**Two examples of neuronal response during match stimulus. (A)** An example of the match stimulus response. The raster plots and spike density plots (σ = 10 ms) are aligned to the onset of the match stimulus (line at time 0). Horizontal axis shows the time from the onset of the match stimulus. Vertical axis shows the firing rate. **(B)** Another example of the recorded neuronal responses. The same convention as in **(A)**.

**Figure 5:**
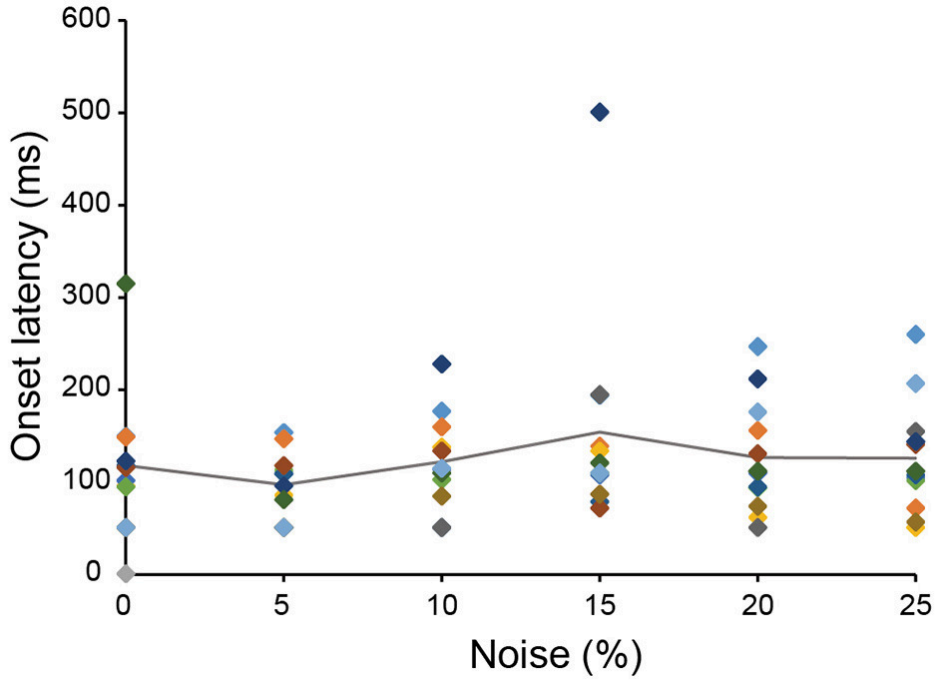
**The relation between neuronal latency and noise level**. The onset latency measured from the spike activity recorded during the match stimulus presentation was plotted against noise level. The same color square means the data from the same neuron. The line shows the averaged onset latency from 13 neurons. Color that indicates data of each neuron matches the color shown in Figure 3.

The 13 neurons in area TE showed selective responses to the 8 match stimuli in 0% noise level (Figure 3). Figure 4 shows 2 examples of neuronal responses to each match stimulus. The neuron in Figure 4A showed the strongest response at pattern 5 in 0% noise level, and the response strength decreased at higher noise levels. The neuron in Figure 4B showed stronger responses at specific patterns in various noise levels. 7/13 neurons showed responses like those seen in Figure 4A, whereas the remaining 6 had their highest activities when there was visual noise. Figure 5 shows that the neuronal latency across neurons to the match stimulus did not change significantly (Kruskal-Wallis rank sum test, *df* = 5, *p* = 0.7267) as a function of noise level.

### The mutual information to discriminate patterns

**Figure 6:**
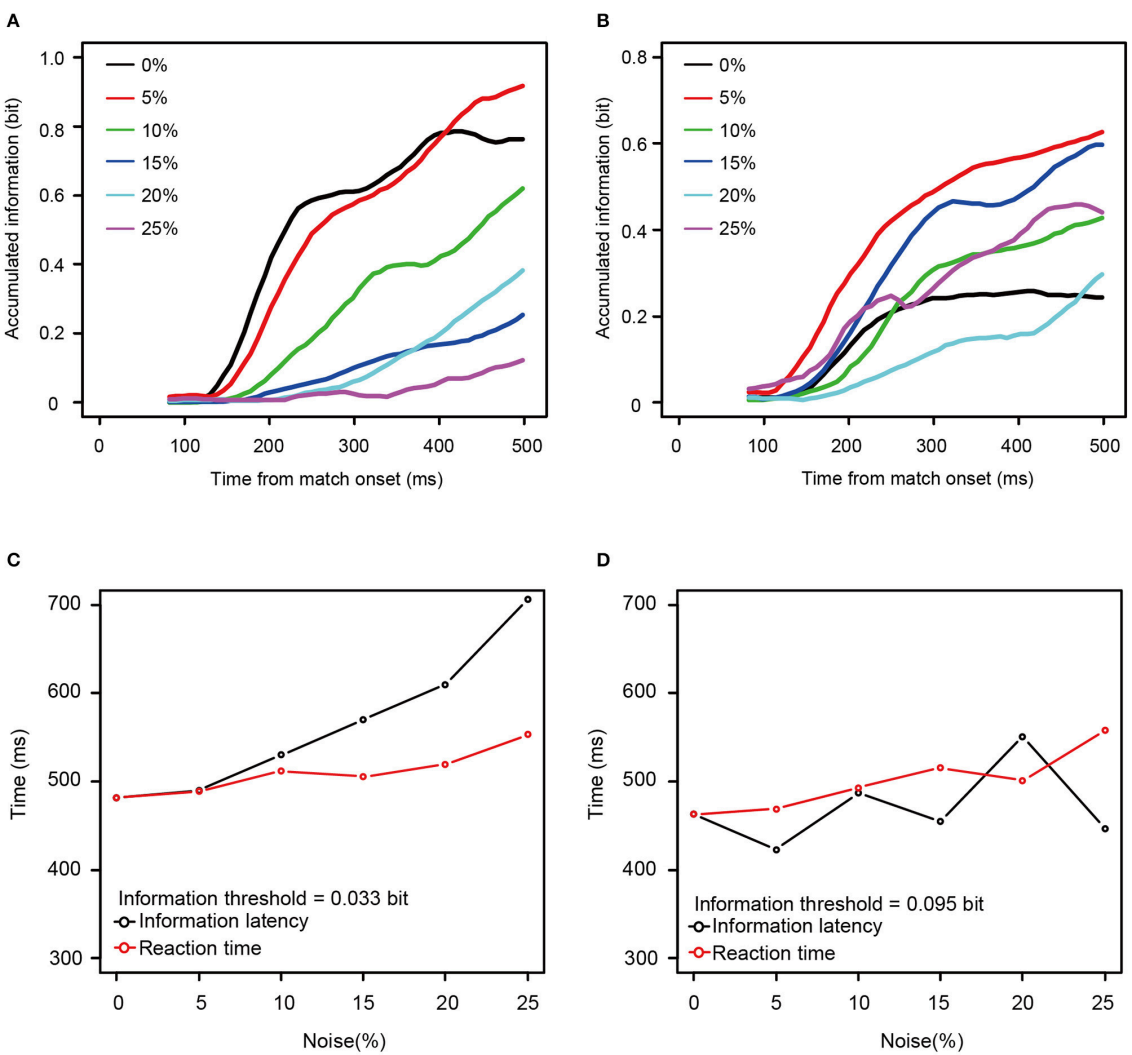
**Two examples of accumulation of information carried by single neurons. (A,B)** Accumulation curve of mutual information (calculated from the same neuron as shown in Figures 4A,B, respectively). Horizontal axis shows the time from the onset of match stimulus. Vertical axis shows accumulated information calculated from neuronal responses using the time window between 50 ms after the onset of match stimulus and the value of the horizontal axis. Black line indicates the information accumulation curve of 0% noise level. Red, green, blue, aqua blue, and purple lines indicate those of 5, 10, 15, 20, and 25% noise levels, respectively. Accumulation curves were smoothed by using simple moving average. **(C,D)** The time required to reach the arbitrary threshold of information (information latency) against noise levels (black circle and line) and the median reaction times against noise levels (red circle and line). The neurons used in **(C,D)** corresponded to those in **(A,B)**, respectively. Horizontal axis shows noise level. Vertical axis shows information latency and the reaction time. In **(C,D)**, the constant values (336 and 261 ms, respectively) were added to the information latencies so that those at the 0% noise level matches with the reaction time at the 0% noise level.

To determine whether the information available for discriminating among stimulus patterns was related to the increase in the behavioral reaction times, we calculated how the information carried by the spike counts grew over time. Figures 6A,B shows the temporal dynamics of information after the onset of match stimulus calculated from the neurons shown in Figures 4A,B. For the neuron taken from Figure 4A, the rate at which the information accumulated rose as a function of the noise level, with information accumulating fastest at zero or 5% noise (Figure 6A), whereas for the neuron taken from Figure 4B, this was not the case.

**Figure 7:**
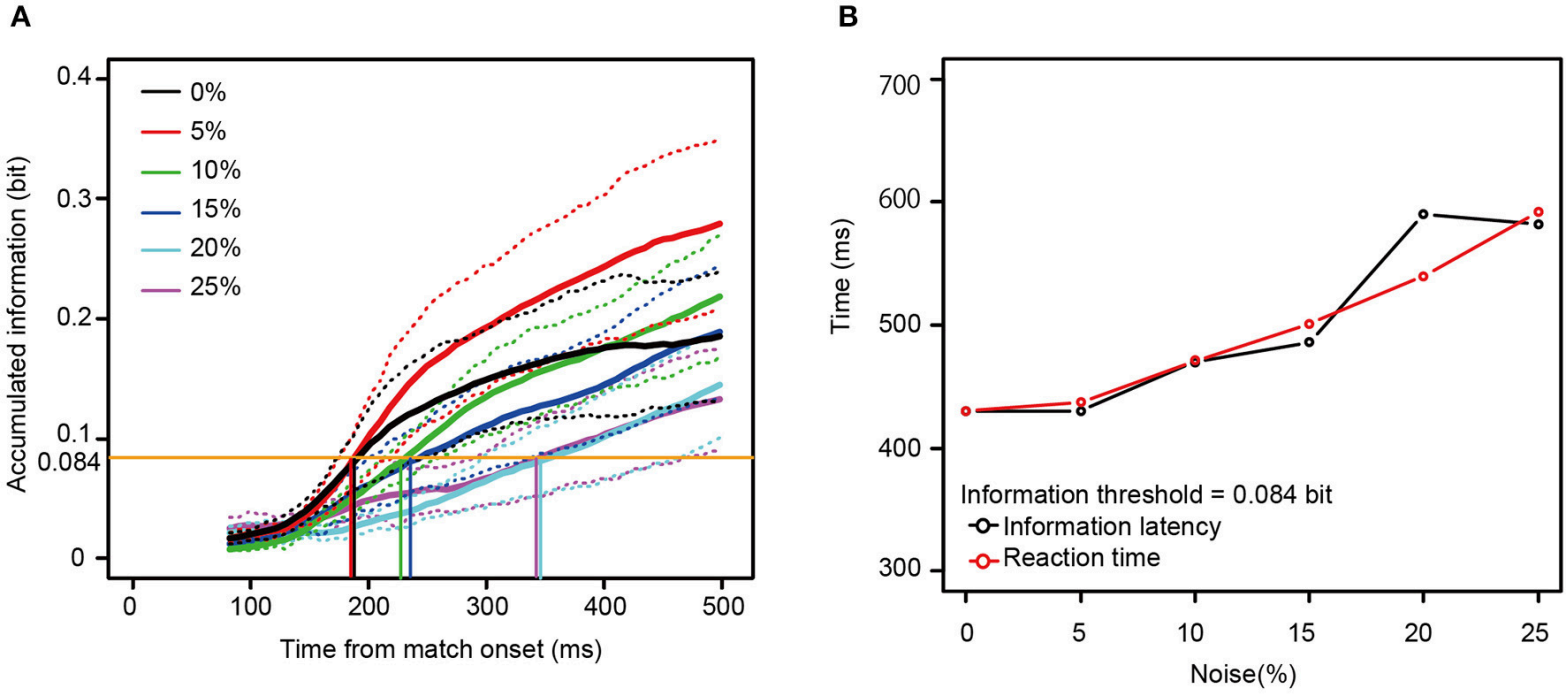
**(A)** The mutual information averaged across all the 13 neurons. **(B)** A comparison between the information latency for the averaged information **(A)** and the median reaction times across the six noise levels. The same convention as in Figure 6 is used here, except that the orange horizontal line in **(A)** means the information threshold of 0.084 bit and 220 ms was added to the information latency. The dotted lines show mean ± SE.

Mutual information rose as a function of time for all 13 neurons for all noise levels. To examine the relationship between the reaction times and the mutual information, we measured the time necessary for the information carried by the neurons to rise to an arbitrary threshold (information latency), using the best fit between the behavioral reaction times and information latency determined by least squares. As shown in Figures 6C,D, the information latency and the reaction times did not match for the neurons in Figure 4. However, when fit for the mutual information averaged across all 13 neurons, the information accumulating at a threshold of 0.084 bits across each noise level seems to be similar to the behavioral reaction times. The time to the threshold of 0.084 bits (the orange horizontal line in Figure 7A) closely matched the behavioral reaction times, with the behavioral reaction times delayed by 220 ms (Figure 7B).

## Discussion

Here we have shown, as before, that in a classical delayed match-to-sample task, monkeys will trade time to maintain accuracy in identifying the match stimulus, when the match stimulus is degraded by visual noise (Shidara and Richmond, 2005). The amount of noise was capped at 25%. It appears that, at this level of noise, the trade-off of time for information allows compensation, that is, the monkeys' error rates increased slightly as the noise level increased although reaction time was more sensitive to the noise. In our neuronal recordings, information about the identity of the visual patterns rises more slowly over time when there is more noise, seeming to show that the neuronal processing integrates stimulus-specific information as time passes, even though the amount of noise on the stimulus unchanged. Thus, the processing acts as if information is progressively interpolated across the noise. This happens even though the onset latency for neuronal responses did not differ across noise levels, implying the visual noise has an effect making it appear as if the visual noise adds noise to the stimulus-elicited responses.

The average amount of information needed to reach the decision threshold was 0.084 bits, with time to reach that threshold being about 180 ms (0 and 5% noise) to about 350 ms (25% noise) after the appearance of the match stimulus, similar to earlier reports (Hung et al., 2005). The information encoded by one neuron was not sufficient for explaining the monkey's behavior, i.e., the reaction times. Thus, the neuronal population in area TE seems to generalize across stimulus degradation in a manner that closely parallels the monkey's ability to recognize the stimulus. This result is consistent with the idea that pattern recognition is processed by neuronal populations (Dicarlo et al., 2012). In addition, Afraz et al. (2006) showed that after microstimulation on the face related area in monkey inferior temporal cortex, the categorization behavior was strongly biased, supporting a central role for the inferior temporal cortex in pattern recognition.

Other recent work (Emadi and Esteky, 2013, 2014) is consistent with our findings, although the results do not match ours in detail. The neural activity they recorded showed increased latencies as visual noise increased, both in a passive task and an active category discrimination task, whereas the neurons we recorded did not change their latencies, but rather showed slower accumulation of stimulus selective information. A number of factors might be responsible for this difference. Our analysis was for single neuronal recordings whereas they recorded multiunit activity; our measurement was information accumulation, which might have different sensitivity to changes in neuronal firing; and finally the tasks were different, ours being stimulus working memory and identification with noise level of up to 25%, theirs being active categorization with up to 60% noise level without a working memory component. Nonetheless, all of the studies support the hypothesis that neural activity in inferior temporal cortex, specifically area TE for us, carries information about stimulus identity that accumulates over time in such a manner that it progressively overcomes the signal degradation imposed by adding visual pixel noise, and that this accumulated information is available for the monkey to monitor as the information rises to a decision threshold.

## Author contributions

BR and MS designed research and conducted experiment. RK conducted data analysis. RK, YS, NM, BR, and MS discussed data. RK wrote the draft of article. RK, YM, NM, BR, and MS revised the manuscript. All authors approved the final version of the manuscript.

### Conflict of interest statement

The authors declare that the research was conducted in the absence of any commercial or financial relationships that could be construed as a potential conflict of interest.
